# Editorial: Precision oncology in checkpoint immunotherapy: leveraging predictive biomarkers for personalized treatment

**DOI:** 10.3389/fimmu.2025.1772930

**Published:** 2026-01-21

**Authors:** Zodwa Dlamini, Abdullah Kahraman, Aristotelis Chatziioannou

**Affiliations:** 1 SAMRC Precision Oncology Research Unit (PORU), DSTI/NRF SARChI Chair in Precision Oncology and Cancer Prevention (POCP), Pan African Cancer Research Institute (PACRI), University of Pretoria, Hatfield, South Africa; 2 Wolfson Wohl Cancer Research Centre, School of Cancer Sciences, University of Glasgow, Garscube Estate, Switchback Road, Bearsden, Glasgow, United Kingdom; 3 School of Veterinary Medicine and Science, University of Nottingham, Nottingham, United Kingdom; 4 Institute for Chemistry and Bioanalytics, School for Life Sciences, University of Applied Sciences Northwestern Switzerland, Muttenz, Switzerland; 5 Swiss Institute of Bioinformatics, Lausanne, Switzerland; 6 Center of Systems Biology, Biomedical Research Foundation of the Academy of Athens (BRFAA), Athens, Greece

**Keywords:** artificial intelligence, immune checkpoint inhibitors, immune microenvironment, multi-omics integration, patient stratification, precision oncology, predictive biomarkers, tumor heterogeneity

## 1 Introduction

Immune checkpoint inhibition has further catalyzed the ongoing decisive shift in oncology from empiricism to biological precision. Yet the heterogeneity of clinical benefit underscores the need for integrative frameworks that resolve how tumor intrinsic programs, the immune microenvironment, and patient-level context collectively govern therapeutic susceptibility. This Special Topic provides convergent evidence for a multimodal biomarker ecosystem across mechanistic biology, cross-cancer multi-omics modeling, artificial intelligence, digital biomarkers, and translational studies and illuminates principles rather than recapitulate study-specific results. We aim to articulate how composite biomarker architectures spanning genomic and epigenomic variation, immune ecologic features, liquid and pathomic readouts, and patient-reported metrics can be operationalized for robust patient stratification and adaptive treatment navigation. In doing so, we foreground methodological rigor, generalizability across tumor types, and equity in access to biomarker testing. The sections that follow move from molecular determinants, through system-level signatures and computational innovations, to microenvironmental architecture and clinical translation, and conclude by outlining pragmatic horizons for embedding multimodal biomarkers into real-time oncologic decision making.

## 2 Molecular determinants of immunotherapy response: regulatory genes, mutation burden, and neoantigen landscape

Several studies in this Research Topic deepen our mechanistic grasp of how tumor-intrinsic factors shape Immune Checkpoint Inhibitors (ICI) responsiveness. Li et al., identified USP38 as an important regulator of malignant progression, adding to the growing group of deubiquitinating enzymes implicated in immune escape and tumor aggressiveness. Niu et al. reveal diagnostic and immunological relevance of TRIT1 expression in hepatocellular carcinoma, connecting tRNA modification pathways with immune infiltration patterns.

Kondrateva et al., offered a significant cross-cancer analysis demonstrating that tumor mutational burden (TMB) can reliably predict neoantigen profiles and forecast immunotherapy response even in microsatellite-stable (MSS) tumors, traditionally considered less immunogenic. These insights emphasize the importance of mutation-derived antigenicity and quality of neoantigens, not only their quantity.

Similarly, Wang et al. (1), characterized radiation-resistance-related genes in pancreatic cancer, revealing transcriptional signatures associated with poor immune activation and decreased treatment efficacy. Zhang et al., uncovered an immunosuppressive role of TSHZ3 in lung adenocarcinoma, illustrating how transcription factors can reshape the tumor microenvironment (TME) to blunt anti-tumor immunity. Collectively, these mechanistic studies reinforce the importance of integrated molecular biomarkers for precision immunotherapy.

## 3 Multi-omics signatures and cross-cancer predictive models: toward universal biomarker frameworks

The Special Topic showcases the power of large-scale multi-omics and pan-cancer analytics. Wu et al., introduced an autonomic-nervous-system (ANS) development-related signature that stratifies immunotherapy response across 30+ cancer types, an innovative intersection of neurobiology and oncology. Zhu et al., conducted a comprehensive analysis of semaphorin family genes in colorectal cancer, demonstrating how neuro-immune signaling molecules contribute to ICI responsiveness, prognosis, and TME remodeling.

Langfelder et al, established a gene signature predicting immunotherapy outcomes in metastatic urothelial carcinoma, while Zheng et al., proposed the α-FAte score as a prognostic model for locoregional immunotherapy in hepatocellular carcinoma. Meanwhile, Zhang et al., evaluated the prognostic value of the Charlson comorbidity index for immunotherapy survival in pancreatic cancer, integrating clinical complexity into biomarker science. These studies illustrate how multi-layered biomarker systems, genomic, transcriptomic, clinical, and comorbidity-based, enhance precision in patient selection.

## 4 AI, machine learning, and digital biomarkers: transforming the predictive landscape

Artificial intelligence continues to revolutionize precision oncology, enabling the extraction of clinically meaningful patterns from high-dimensional datasets. Several contributions to this Research Topic illustrate its transformative potential. Shen et al., applied a machine-learning quality-of-life (QoL) scale to predict immunotherapy response in advanced NSCLC patients, one of the first studies integrating patient-reported outcomes into predictive modeling for ICIs. Wan et al., combined pathomics with deep learning to improve subtyping of uveal melanoma, identifying high-risk immune infiltration profiles linked to poor prognosis. Xie et al, introduced a dynamic peripheral blood-based model for ICI responsiveness in lung squamous cell carcinoma, advancing the field of liquid-immune monitoring.

Single-cell transcriptomics also features prominently. Wan et al., identified CTHRC1 as a driver of invasion and immunosuppressive TME in triple-negative breast cancer, demonstrating how single-cell resolution can uncover previously obscured drivers of immune evasion. Together, these innovations signal the arrival of computational precision immunology as a central pillar of treatment personalization.

## 5 Tumor microenvironment architecture: immune infiltration, TLS dynamics, and stromal modulation

The TME remains a critical determinant of immunotherapy success. Li et al., reported that the presence of germinal center–like tertiary lymphoid structures (GC-TLS) after neoadjuvant chemo-immunotherapy predicts disease progression in lung squamous cell carcinoma. Their findings align with emerging evidence that TLS formation serves as a surrogate for anti-tumor immune activation.

Bida et al. presented a comprehensive synthesis of tumor-infiltrating lymphocytes (TILs) in melanoma, bridging prognostic relevance with therapeutic applications. This aligns with growing global interest, as highlighted by Bao et al.‘s analysis of worldwide research trends in TLS from 2014–2023.

Li et al., construct a novel PNI-based nomogram to predict immunotherapy efficacy in advanced breast cancer, revealing how nutritional-immune interplay correlates with ICI response. Tang et al., evaluated the ratio of inhibitory-to-stimulatory immune checkpoints in breast cancer, providing a quantitative framework to evaluate immunological tone within the TME. These contributions underscore the necessity of integrated immune and stromal biomarkers for refining precision strategies.

## 6 Translational and clinical evidence: from ctDNA monitoring to combined modality trials

Clinical translation remains a major theme within the Special Topic. Zhang et al., compared cohorts to evaluate ctDNA-guided adjuvant therapy de-escalation in head and neck squamous cell carcinoma, addressing a key challenge of overtreatment and treatment-related toxicities. Cui et al., systematically review neoadjuvant immunotherapy in mismatch repair-deficient colorectal cancer, summarizing response rates and histological regression patterns.

Pu et al. provided a meta-analysis demonstrating the efficacy and safety of PD-1/PD-L1 inhibitors combined with chemotherapy in advanced gastric and gastroesophageal junction cancers. Zhang et al., conducted a network meta-analysis evaluating monotherapy versus combination strategies in renal cell carcinoma, offering comparative evidence for clinicians navigating complex therapeutic choices. Two highly insightful case reports complement these studies. Feng et al., described a noteworthy response to front-line cadonilimab plus chemotherapy in lung adenocarcinoma with STK11 mutation, typically associated with ICI resistance. Da et al. document a significant response in AFP-producing gastric cancer treated with combined chemotherapy and immunotherapy.

These clinical insights emphasize the importance of real-world data, biological nuance, and disease-specific considerations when applying precision oncology principles.

## 7 Future horizons: convergence of biology, computation, environment, and patient-centered care

**Figure 1:**
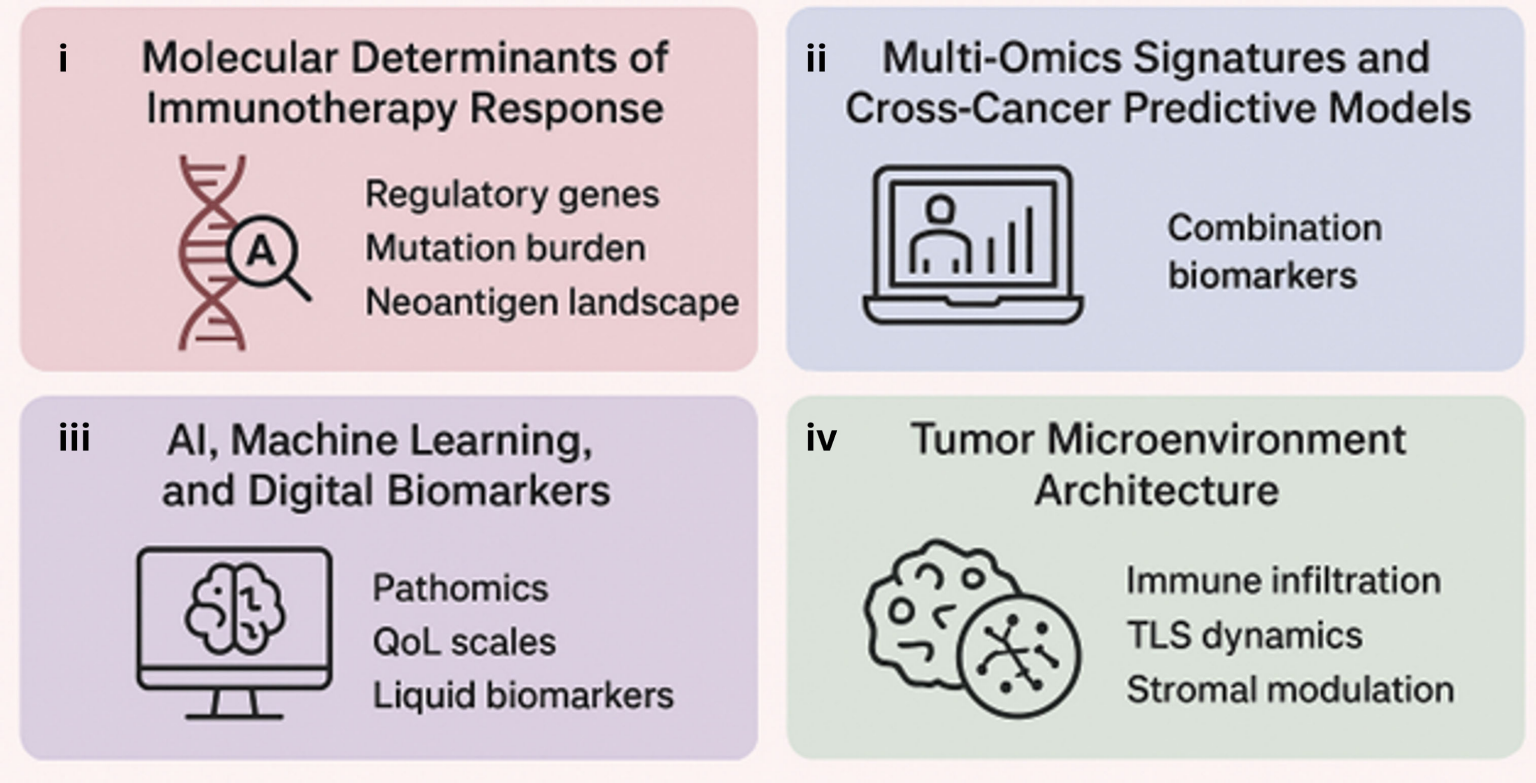
Precision oncology framework for checkpoint immunotherapy. This schematic illustrates an integrated precision oncology approach to ICI therapy, emphasizing the role of predictive biomarkers in personalizing cancer treatment. The framework is organized into four interrelated domains: (i) Molecular determinants of immunotherapy response, including regulatory gene alterations, tumor mutational burden, and the neoantigen landscape that influence tumor immunogenicity; (ii) Multi-omics signatures and cross-cancer predictive models, highlighting the integration of genomic, transcriptomic, proteomic, and epigenomic data to derive combination biomarkers applicable across tumor types; (iii) AI, machine learning, and digital biomarkers, encompassing pathomics, patient-reported QoL scales, and liquid biomarkers to enable dynamic and non-invasive response prediction; and (iv) Tumor microenvironment architecture, focusing on immune cell infiltration, TLS dynamics, and stromal modulation that collectively shape therapeutic outcomes. Together, these domains underscore the multidimensional data integration required to optimize patient stratification and improve clinical responses to checkpoint immunotherapy.

Across all contributions, a central message emerges that precision immunotherapy requires the integration of diverse molecular, clinical, ecological, computational, and patient-derived (**Figure 1**) biomarkers. Environmental influences, ancestry-related factors, exposomic variables, and socioeconomic determinants of health are increasingly recognized as modifiers of immunotherapy outcomes and should be included in future predictive frameworks (**Figure 1**).

The Special Topic demonstrates that the next frontier in precision oncology is not a single biomarker, but a holistic, systems-level multimodal biomarker ecosystems. As we move forward, several key directions stand out:

1. Integration of multi-omics in real-time clinical workflows.

Including adaptive biomarker updating during treatment.

2. Fine-tuning of AI with Biological Complexity at different scales.

This entails robust integration of network biology tenets and results in AI-logic.

3. Expansion of AI-driven predictive modelling

Particularly in underrepresented populations globally.

4. Incorporation of immune ecology and tissue architecture.

Especially TLS, TIL patterns, and dynamic immune remodeling.

5. Greater emphasis on liquid and digital biomarkers.

Enabling early detection of resistance and treatment adaptation.

6. Prioritization of equitable access to biomarker testing.

Ensuring global relevance and reducing disparities in cancer immunotherapy.

Together, these articles provide a foundational contribution to the future of precision immunology, offering not only mechanistic insights but also practical tools for clinical translation.

## 8 Conclusion

The contributions in this Special Topic collectively underscore that precision immunotherapy is no longer defined by single biomarkers but by integrated, multimodal frameworks that unite molecular determinants, multi-omics signatures, computational analytics, and immune-ecological profiling. Advances in artificial intelligence, liquid and digital biomarkers, and real-world clinical evidence are accelerating the transition from population-level strategies to adaptive, patient-centered care. Future progress will hinge on embedding these composite biomarker systems into routine workflows, ensuring methodological rigor, global equity, and dynamic updating during treatment. By converging biology, computation, and patient context, precision oncology stands poised to deliver more predictable, durable, and equitable outcomes in checkpoint immunotherapy.
